# Measuring approach–avoidance tendencies towards food with touchscreen-based arm movements

**DOI:** 10.1007/s00426-019-01195-1

**Published:** 2019-05-04

**Authors:** Adrian Meule, Anna Richard, Anja Lender, Radomir Dinic, Timo Brockmeyer, Mike Rinck, Jens Blechert

**Affiliations:** 1 Department of Psychology, University of Salzburg, Hellbrunner Straße 34, 5020 Salzburg, Austria; 2 Center for Cognitive Neuroscience, University of Salzburg, Salzburg, Austria; 3 Schoen Clinic Roseneck, Prien am Chiemsee, Germany; 4 Department of MultiMedia Technology, Salzburg University of Applied Sciences, Puch, Austria; 5 Institute of Psychology, University of Goettingen, Göttingen, Germany; 6 Behavioural Science Institute, Radboud University Nijmegen, Nijmegen, The Netherlands

## Abstract

Most tasks for measuring automatic approach–avoidance tendencies do not resemble naturalistic approach–avoidance behaviors. Therefore, we developed a paradigm for the assessment of approach–avoidance tendencies towards palatable food, which is based on arm and hand movements on a touchscreen, thereby mimicking real-life grasping or warding movements. In Study 1 (*n* = 85), an approach bias towards chocolate-containing foods was found when participants reached towards the stimuli, but not when these stimuli had to be moved on the touchscreen. This approach bias towards food observed in grab movements was replicated in Study 2 (*n* = 60) and Study 3 (*n* = 94). Adding task features to disambiguate distance change through either corresponding image zooming (Study 2) or emphasized self-reference (Study 3) did not moderate this effect. Associations between approach bias scores and trait and state chocolate craving were inconsistent across studies. Future studies need to examine whether touchscreen-based approach–avoidance tasks reveal biases towards other stimuli in the appetitive or aversive valence domain and relate to relevant interindividual difference variables.

**Electronic supplementary material:**

The online version of this article (10.1007/s00426-019-01195-1) contains supplementary material, which is available to authorized users.

## Introduction

Humans typically approach appetitive stimuli and try to avoid aversive ones. To bypass limitations of self-report measures of such partially automatic approach–avoidance tendencies towards environmental stimuli, several behavioral reaction time tasks have been developed. Two prominent examples include manikin tasks, in which a symbol of a person needs to be moved towards or away from the target stimulus (de Houwer, Crombez, Baeyens, & Hermans, 2001), and joystick-based tasks, in which the target stimulus needs to be moved towards or away from oneself by pulling or pushing a joystick (Rinck & Becker, 2007).

While such tasks represent established and relatively well-validated tools for measuring approach–avoidance tendencies, studies that aimed at demonstrating an approach bias towards (high-calorie) food produced mixed findings. Specifically, the majority of studies that used joystick-based tasks did not demonstrate an approach bias towards (high-calorie) food relative to reactions to control stimuli or found such a bias only in certain subgroups of participants (Brockmeyer, Hahn, Reetz, Schmidt, & Friederich, 2015; Kakoschke, Kemps, & Tiggemann, 2015; Maas, Keijsers, et al., 2017; Maas, Keijsers, Rinck, Tanis, & Becker, 2015; Maas, Woud, et al., 2017; Machulska, Zlomuzica, Adolph, Rinck, & Margraf, 2015; Paslakis, Kühn, Grunert, & Erim, 2017; Paslakis et al., 2016). We previously demonstrated an approach bias towards chocolate-containing food in a predominantly young, female sample, but this bias was only found when stimulus categories (food vs. objects) were explicitly associated with approach–avoidance instructions (Lender, Meule, Rinck, Brockmeyer, & Blechert, 2018).

One reason for these inconsistencies may be that approaching or avoiding real items such as food involves reaching, grasping, and moving these objects, which are movements that are not executed during the above-mentioned computerized tasks. Recent research has started to examine how to measure approach–avoidance tendencies based on more naturalistic movements. Eerland, Guadalupe, Franken, and Zwaan (2012), for example, used posture on a balance board as an index for approach–avoidance behaviors. Recently, Schroeder, Lohmann, Butz, and Plewnia (2016) demonstrated an approach bias towards food based on grasping hand movements in a virtual reality setting. Shen, Zhang, and Krishna (2016) have also demonstrated the relevance of directly, naturalistically interacting with food stimuli in a food choice task. In their studies, participants performed the same task either on a touchscreen device or on a desktop computer. The authors found what they termed a “direct-touch effect” such that participants who performed the task on a touchscreen made more hedonic food choices than those who performed the task with a non-touch interface. Finally, Zech and colleagues implemented an approach–avoidance task (AAT) on a smartphone that could be moved closer or further away to simulate naturalistic grasping or rejection movements (Cring, 2017; Zech, 2015).

Due to the popularity and wide availability of touchscreen-based devices such as smartphones and tablet computers, we have recently examined an implementation of an AAT on a touchscreen monitor (Meule, Lender, Richard, Dinic, & Blechert, 2019). Here, participants had to pull or push pictures of chocolate-containing foods or objects towards or away from themselves by dragging the pictures to the top or bottom of a screen, which was horizontally positioned in front of them. An approach bias towards these foods, however, was only found in individuals who reported that they frequently crave chocolate. Moreover, while this paradigm represents an AAT with more naturalistic arm movements in terms of pushing and pulling the pictures, it still deviates from real-life approach and avoidance behaviors. For example, approaching food may involve reaching towards that food first and then pulling it closer and avoiding food may involve first grabbing it and then moving it away from oneself. Therefore, we modified our previous touchscreen-based paradigm such that both grabbing and dragging movements would be required.

In the current studies, we thus tested a paradigm in which participants had to lean forward and reach out to distal stimuli and then drag these stimuli towards themselves (i.e., approach) and to grasp proximal stimuli and then drag these stimuli away from themselves (i.e., avoidance) on a touchscreen monitor. Through this setup, we were able to differentiate between the time participants needed to reach the target stimulus (grabbing time) and the time participants needed to move the stimulus towards or away from themselves (dragging time). Across three studies, we used pictures of chocolate-containing foods and pictures of non-edible objects, which we also employed in our previous studies (Lender et al., 2018; Meule et al., 2019). We expected that participants in the present studies would show an approach bias towards food (e.g., shorter reaction times in pull food than pull objects trials) in our touchscreen-adapted task. This bias may be reflected in grab movements (which would be in line with the findings by Schroeder et al., 2016), in drag movements (which would correspond to manikin or joystick tasks, where no grab movements are required), or both.

In Study 1, participants were instructed to respond to either pictures of food or objects by reaching towards them and moving them to the opposite side of the screen. That is, when target stimuli were displayed near the top (distal) edge of a horizontally positioned touchscreen, they had to be pulled (approached) and when target stimuli were displayed at the bottom (proximal) edge of the screen, they had to be pushed (avoided). In Study 2, we examined whether introducing a zooming effect would facilitate approach–avoidance inclinations. As in joystick tasks (Rinck & Becker, 2007), the picture would enlarge in pull trials and would shrink in push trials. In Study 3, we examined whether manipulating self-reference would modulate approach–avoidance movements. Specifically, we tested whether presenting a manikin (representing the participant) at the bottom half of the screen would facilitate approach–avoidance inclinations and whether presenting a manikin at the top half of the screen would reverse response patterns. As approach bias towards food was related to higher trait or state food craving in previous studies (Brockmeyer et al., 2015; Lender et al., 2018; Meule et al., 2019), we explored whether trait chocolate craving as well as state chocolate craving before and after the task were associated with approach–avoidance tendencies across all three studies. Although relationships between craving and approach biases have not been consistently found in the literature, positive relationships may provide an indication of convergent validity of our new paradigm.

## Study 1

### Methods

*Participants* Eighty-five individuals (82.4% female, *n* = 70) participated in this study. Mean age was 22.1 years (SD = 2.63) and mean body mass index was 22.5 kg/m^2^ (SD = 3.23). Mean hunger ratings on a scale from 0 = *not hungry at all* to 10 = *very hungry* were 4.66 (SD = 2.69). Thirty participants (56.7% German, *n* = 17; 40.0% Austrian, *n* = 12; 3.30% other citizenship, *n* = 1) were tested at the University of Salzburg, Austria. Fifty-five participants (96.4% German, *n* = 53; 3.60% other citizenship, *n* = 2) were tested at the Radboud University Nijmegen, The Netherlands. Participants’ sex (χ_(1)_^2^ = 0.03, *p* = 0.861), hunger ratings, and age (both *t*s < 1.26, *p*s > 0.211) did not differ between the two study centers.

*AAT* The AAT included 16 pictures displaying chocolate-containing foods and 16 pictures displaying non-edible objects, which were obtained from the food-pics database (Blechert, Meule, Busch, & Ohla, 2014). Picture numbers in the food-pics database are 004, 079, 107, 111, 137, 140, 162, 163, 165, 168, 189, 286, 289, 465, 500, 510 (chocolate pictures), and 1004, 1015, 1045, 1056, 1059, 1095, 1146, 1188, 1212, 1226, 1227, 1260, 1265, 1279, 1283, 1293 (neutral pictures). Food and objects pictures did not differ in color, size, brightness, contrast, complexity, recognizability, or familiarity (all *t*s < 1.27, *p*s > 0.214). Each picture had a resolution of 96 dpi (600 × 450 pixels). The pictures have been previously used in a joystick-based AAT (Lender et al., 2018).

**Fig. 1:**
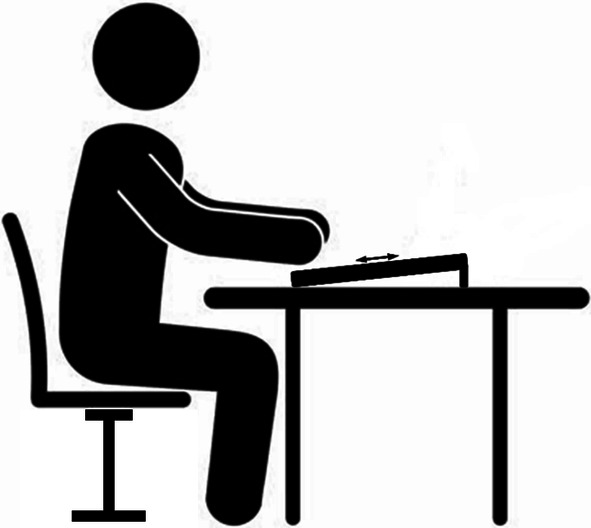
Experimental setup in all three studies. Participants sat in front of a table on which a touchscreen monitor was positioned in portrait orientation with an angle of approximately 15°

**Fig. 2:**
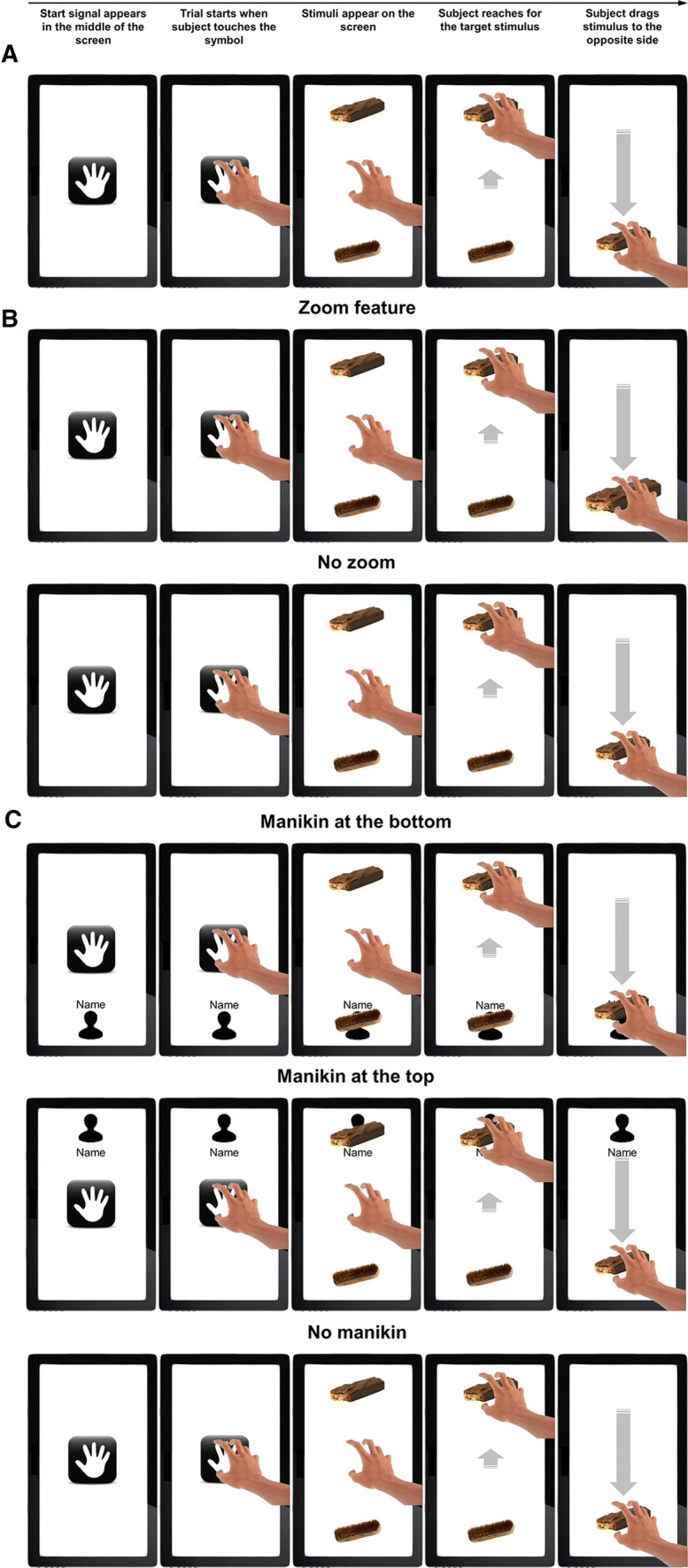
Representative pull trials in a food block in Study 1 (**a**), Study 2 (**b**), and Study 3 (**c**). Each trial began with the display of a hand symbol in the center of the screen. When participants touched this symbol with five fingers, two pictures appeared at the top and bottom of the screen. Participants were instructed to either respond to pictures with food or to pictures with non-edible objects and to move pictures at the top towards themselves (to the bottom of the screen) and to move pictures at the bottom away from themselves (to the top of the screen). The picture disappeared and the next trial started when the picture reached the opposite border of the screen. In Study 1, all participants performed the same task (**a**). In Study 2, one group of participants performed the task with a zoom feature and one group of participants performed the task as in Study 1 (**b**). In Study 3, one group of participants performed the task with a manikin displayed at the bottom, one group of participants performed the task with a manikin displayed at the top, and one group of participants performed the task as in Study 1 (**c**). Note that the arrows were not used in the task but are presented here for illustration

The task was programmed in unity (https://unity3d.com) and displayed on a 23-inch iiyama ProLite T2336MSC-B2 touchscreen monitor with a resolution of 1920 × 1080 pixels. Participants were seated in front of a table on which the touchscreen monitor was positioned in portrait orientation with an angle of approximately 15° relative to the horizontal table top (Fig. 1). Each trial started with presentation of a hand symbol in the center of the screen. When participants placed five fingers on the hand symbol, two pictures simultaneously appeared on the screen, which were either a food picture on the top (distal part of the screen) and an object picture on the bottom (proximal part of the screen) or vice versa (Fig. 2a). Participants were instructed to reach for the target picture. Instructions then read “When the [target] picture is in the lower half [of the screen], push it away from yourself. When the [target] picture is in the upper half [of the screen], pull it towards you.”. When participants reached the target picture, the other picture disappeared so that the target picture could be moved to the other side of the screen. When participants reached to the wrong picture, this picture could not be moved (i.e., a trial could only be completed by grabbing the correct picture and moving it to the other side of the screen). The task consisted of two blocks and participants were instructed to respond to the food pictures in one block and to the objects pictures in the other block. Block order was counterbalanced across participants. Within each block, each picture was presented four times at the top and four times at the bottom (in randomized order), totaling 128 trials in one block. Thus, the task consisted of 256 trials in total, and participants had to pull food, push food, pull objects, and push objects in 64 trials each.

*Questionnaires* The German, chocolate-adapted version of the Food Cravings Questionnaire–Trait–reduced (FCQ–T–r; Meule & Hormes, 2015) was used to measure the frequency and intensity of chocolate cravings in general. The scale has 15 items which are scored from 1 = *never* to 6 = *always*. Internal reliability was *α* = 0.961 in the current study. The German, chocolate-adapted version of the Food Cravings Questionnaire–State (FCQ–S; Meule & Hormes, 2015) was used to measure the intensity of current chocolate craving and hunger before and after the AAT. The scale has 15 items (12 items for the chocolate craving subscale and 3 items for the hunger subscale) which are scored from 1 = *strongly disagree* to 5 = *strongly agree*. Internal reliabilities of the craving subscale were *α* = 0.939 before and *α* = 0.950 after the task. Internal reliabilities of the hunger subscale were *α* = 0.902 before and *α* = 0.929 after the task.

*Procedure* The study was approved by the institutional review board of the University of Salzburg. Participants were recruited and tested at the University of Salzburg and at Radboud University. A few days prior to the laboratory testing session, participants completed an online survey, which included the FCQ–T–r. In the laboratory testing session, participants signed informed consent, provided the sociodemographic information, and completed the FCQ–S. They then performed the AAT and, subsequently, completed the FCQ–S again. Participation was reimbursed with course credits.

*Data analyses* Trials in which participants lifted their hand too early or reached to the wrong picture were excluded from analyses (4.25% of all trials). We differentiated two types of reaction times: the time between picture onset until participants reached the target stimulus (*grabbing time*) and the time participants needed to move the target stimulus to the border of the screen (*dragging time*). Bootstrapped split-half reliability estimates for each condition (pull food, push food, pull objects, push objects) were obtained using the *average* function of the R package *splithalf* version 0.5.2 (Parsons, 2018) performing 5000 random splits. Reliability estimates were *r* = 0.90–0.91 (Spearman–Brown-corrected *r*_sb_ = 0.95–0.96) for grabbing time and *r* = 0.98 (Spearman–Brown-corrected *r*_sb_ = 0.99) for dragging time. In line with joystick-based AAT studies (Rinck & Becker, 2007), median reaction times of all trials as a function of condition were calculated for each participant. Analyses of variance for repeated measures with *trial type* (pull vs. push) and *stimulus* (food vs. objects) as within-subjects factors were run separately for grabbing times and dragging times. To examine correlates of AAT performance, approach bias scores were calculated separately for grabbing time and dragging time (approach bias score = [reaction time for pushing food − reaction time for pulling food] − [reaction time for pushing objects − reaction time for pulling objects]). Thus, positive values indicate an approach bias towards chocolate-containing food and negative values indicate an avoidance bias from chocolate-containing foods, relative to non-edible objects. For this approach bias score, reliability estimates using the *difference*-*of*-*difference* function of *splithalf* were *r* = 0.50 (Spearman–Brown-corrected *r*_sb_ = 0.66) for grabbing time and *r* = 0.43 (Spearman–Brown-corrected *r*_sb_ = 0.59) for dragging time.

### Results

**Fig. 3:**
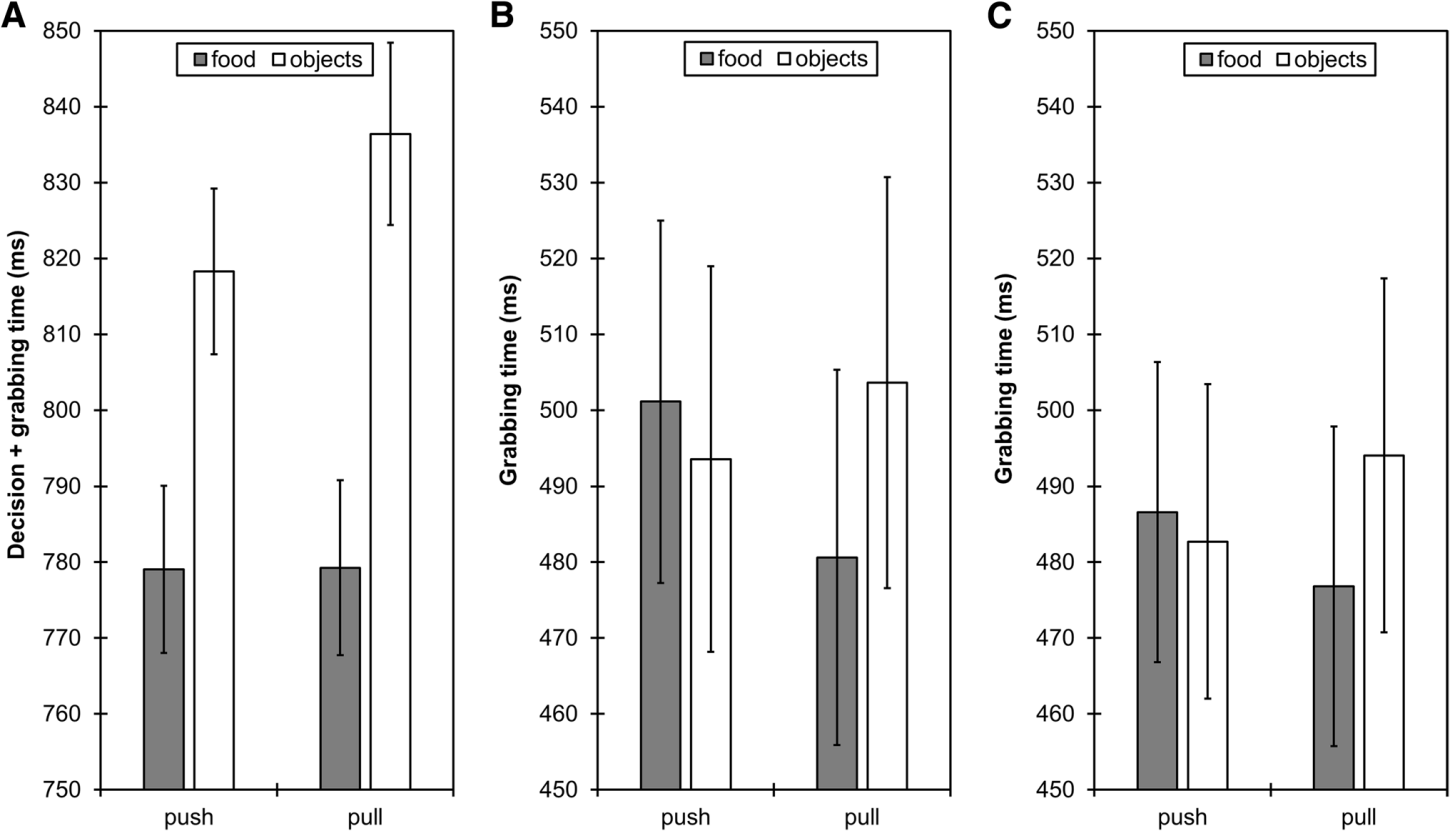
Grabbing times as a function of trial type (push vs. pull) and stimulus (food vs. objects) in Study 1 (**a**), Study 2 (**b**), and Study 3 (**c**). Note that grabbing times in Study 1 include the time participants needed to recognize the pictures and decide to which picture they had to reach (i.e., the time between picture onset and the moment when participants lifted their hand off the starting position). This decision time was not included in grabbing times in Study 2 and Study 3. Therefore, grabbing times are longer in Study 1 than in Study 2 and Study 3 and include a main effect of stimulus (i.e., that participants were faster for food than objects across trial types), which was similarly found in Study 2 and Study 3 when decision time was analyzed separately. Error bars represent standard errors of the mean

*Grabbing time* A significant main effect of *stimulus* [*F*_(1,84)_ = 56.7, *p* < 0.001, *η*_p_^2^ = 0.403] indicated that participants reacted faster to food (*M* = 779 ms, SD = 96.8) than to objects (*M* = 827 ms, SD = 95.9). This effect, however, was qualified by a significant interaction *trial type* × *stimulus* [*F*_(1,84)_ = 5.45, *p* = 0.022, *η*_p_^2^ = 0.061]. Following up this interaction effect with paired *t* tests was inconclusive, as grabbing times in pull versus push trials were not significantly different for either stimulus category (both *t*s < 1.87, *p*s > 0.065) and were faster for food versus objects in both pull and push trials (both *t*s > 5.26, *p*s < 0.001). The pattern of the means, however, suggests that grabbing objects in pull trials was slightly slower than in push trials, potentially due to basic motor movement characteristics (i.e., more shoulder muscle activity necessary when reaching towards the distal side of the touchscreen). Taking this object-related movement pattern as a reference, this slowing was not observed for grabbing food in pull versus push trials, which might hint at a facilitation of the grab movement in pull trials due to food approach (Fig. 3a). The main effect of *trial type* was not significant [*F*_(1,84)_ = 1.26, *p* = 0.265, *η*_p_^2^ = 0.015].

*Dragging time* A significant main effect of *trial type* [*F*_(1,84)_ = 19.4, *p* < 0.001, *η*_p_^2^ = 0.187] indicated that participants were faster in push (*M* = 408 ms, SD = 108) than in pull trials (*M* = 423 ms, SD = 108). The main effect of *stimulus* [*F*_(1,84)_ = 1.57, *p* = 0.214, *η*_p_^2^ = 0.018] and the interaction *trial type* × *stimulus* [*F*_(1,84)_ = 0.80, *p* = 0.373, *η*_p_^2^ = 0.009] were not significant.

*Correlates of approach bias scores* Grabbing time approach bias scores did not correlate with trait chocolate craving, current chocolate craving or hunger before or after the task (all *r*s < − 0.125, *p*s > 0.257). Dragging time approach bias scores did not correlate with current hunger before or after the task (both *r*s < − 0.106, *p*s > 0.337) but correlated positively with trait chocolate craving (*r* = 0.250, *p* = 0.021) and current chocolate craving before (*r* = 0.239, *p* = 0.028) and after the task (*r* = 0.217, *p* = 0.046).

### Conclusion

Study 1 revealed an approach bias towards food as indicated by the *trial type* × *stimulus* interaction. This effect was not found for drag movements and is, therefore, in line with the findings by Schroeder et al. (2016) who examined grasp movements towards food in a virtual reality setting. When examining correlates of approach bias scores, however, higher approach bias scores for drag but not grab movements were related to higher trait and state chocolate craving, in line with findings previous studies (Brockmeyer et al., 2015; Lender et al., 2018; Meule et al., 2019). In sum, although the data seemed promising for a first touchscreen-based implementation of an AAT, there was a need for replication and clarification, which motivated Study 2.

## Study 2

Study 1 revealed an approach bias towards food in grab movements, but post hoc tests when comparing the single conditions were not clear. No approach bias was found for drag movements. Thus, Study 2 examined whether strengthening approach–avoidance associations with the executed arm movements would provide a more clear-cut pattern of results. Most joystick-based AAT implementations use a zooming feedback to facilitate perceiving pull and push movements as approach and avoidance behavior (Laham, Kashima, Dix, & Wheeler, 2015). That is, picture size increases in pull trials and decreases in push trials. This zooming feature might be crucial for the emergence of approach–avoidance biases (Phaf, Mohr, Rotteveel, & Wicherts, 2014). Thus, Study 2 examined whether introducing a zooming effect would produce an approach bias towards food when dragging the pictures on the screen and whether the grabbing bias of Study 1 could be strengthened. For this, we used a between-subjects design where one group of participants performed the same task as in Study 1 and another group of participants performed the task with a zoom feature.

An additional change compared to Study 1 concerns the calculation of reaction times. In Study 1, we calculated grabbing time as the time between picture onset and reaching the target stimulus. However, this conflated the time participants needed to recognize the pictures and decide whether they have to reach to the picture at the top or bottom and the time participants needed to reach to the target stimulus. To remedy this, we differentiated between three reaction times in Study 2: the time between picture onset and the moment when participants lifted their hand off the screen (*decision time*), the time between the moment when participants lifted their hand off the screen and when they reached the target stimulus (*grabbing time*), and—as in Study 1—the time participants needed to move the target stimulus to the border of the screen (*dragging time*). This allowed us to conduct a more fine-grained analysis of action preparation and motor movement execution effects, in line with previous research that indicated that approach–avoidance biases might emerge prior to the execution of the actual motor movement (Rotteveel & Phaf, 2004).

### Methods

*Participants* Sixty women participated in this study at the University of Goettingen, Germany. Mean age was 23.5 years (SD = 2.88) and mean body mass index was 21.3 kg/m^2^ (SD = 2.44). Most participants had German citizenship (93.3% German, *n* = 56; 6.70% other citizenship, *n* = 4). Mean food deprivation (i.e., time since participants’ last meal) was 3.08 h (SD = 3.26).

*AAT* The task and apparatus were equal to Study 1, except that half of participants performed the task that included a zooming effect when dragging the stimuli on the screen (Fig. 2b). In pull trials, picture size increased by 20% during the drag movement and—when the picture reached the border of the screen—picture size increased threefold within 500 ms and disappeared. In push trials, picture size decreased by 20% during the drag movement and—when the picture reached the border of the screen—picture size decreased to zero within 500 ms.

*Questionnaires* As in Study 1, the chocolate version of FCQ–T–r was used to measure trait chocolate craving (*α* = 0.953), and the chocolate version of the FCQ–S was used to measure current chocolate craving (*α* = 0.903 before and *α* = 0.942 after the task) and hunger (*α* = 0.850 before and *α* = 0.899 after the task).

*Procedure* The study was approved by the institutional review board of the Institute of Psychology at the University of Goettingen. Participants were recruited and tested at the University of Goettingen. In the laboratory testing session, participants signed informed consent and completed the FCQ–S. They were then randomly assigned to the AAT either with or without the zooming feature. After the AAT, they completed the FCQ–S again as well as the FCQ–T–r and other questionnaires that are not reported here. Participation was reimbursed with course credits or € 8.

*Data analyses* Participants in the group with (*n* = 30) and without (*n* = 30) the zooming feature did not differ in food deprivation, age, trait chocolate craving, current chocolate craving, or hunger before the task (all *t*s < 0.81, *p*s > 0.423). Trials in which participants lifted their hand too early or reached to the wrong picture were excluded from analyses (4.11% of all trials). Bootstrapped split-half reliability estimates in the four conditions (pull food, push food, pull objects, push objects) were *r* = 0.94–0.96 (*r*_sb_ = 0.97–0.98) for decision time, *r* = 0.95–0.96 (*r*_sb_ = 0.97–0.98) for grabbing time, and *r* = 0.98–0.99 (*r*_sb_ = 0.99) for dragging time. Median reaction times were submitted to analyses of variance for repeated measures with *group* (zoom vs. no zoom) as between-subjects factor, and *trial type* (pull vs. push) and *stimulus* (food vs. objects) as within-subjects factors. Approach bias scores were calculated for each reaction time as in Study 1. Reliability estimates using the *difference*-*of*-*difference* function of *splithalf* were *r* = − 0.26 (*r*_sb_ = − 0.39) for the decision time approach bias score, *r* = 0.41 (*r*_sb_ = 0.58) for the grabbing time approach bias score, and *r* = 0.65 (*r*_sb_ = 0.78) for the dragging time approach bias score.

### Results

*Decision time* A significant main effect of *stimulus* [*F*_(1,58)_ = 11.6, *p* = 0.001, *η*_p_^2^ = 0.167] indicated that participants reacted faster to food (*M* = 337 ms, SD = 153) than to objects (*M* = 382 ms, SD = 161). A significant main effect of *group* [*F*_(1,58)_ = 6.11, *p* = 0.016, *η*_p_^2^ = 0.095] indicated that participants in the group without the zoom feature (*M* = 314 ms, SD = 153) reacted faster than participants in the group with the zoom feature (*M* = 405 ms, SD = 132). No other effects were significant (all *F*s < 2.22, *p*s > 0.142).

*Grabbing time* As in Study 1, the interaction *trial type* × *stimulus* was significant [*F*_(1,58)_ = 6.16, *p* = 0.016, *η*_p_^2^ = 0.096]. Yet again, following up this interaction effect with paired *t* tests was inconclusive as grabbing times in pull versus push trials were not significantly different for either stimulus category (both *t*s < 1.85, *p*s > 0.069) and did not differ for food versus objects in either trial type (both *t*s < 1.52, *p*s > 0.133). Similar to Study 1, however, grabbing objects in pull trials was slightly slower than grabbing objects in push trials and this direction reversed for foods: they were grabbed faster in pull than in push trials at a descriptive level (Fig. 3b). Thus, this crossed interaction again points to an approach bias towards food as reflected in grab movements. A significant main effect of *group* [*F*_(1,58)_ = 5.88, *p* = 0.018, *η*_p_^2^ = 0.092] indicated that participants in the group with the zoom feature (*M* = 439 ms, SD = 174) were faster than participants in the group without the zoom feature (*M* = 550 ms, SD = 179). No other effects were significant (all *F*s < 3.29, *p*s > 0.074).

*Dragging time* There were no significant effects (all *F*s < 0.96, *p*s > 0.331).

*Correlates of approach bias scores* Decision time and grabbing time approach bias scores did not correlate with trait chocolate craving, current chocolate craving or hunger before or after the task (all *r*s between − 0.169 and 0.138, *p*s > 0.195). Dragging time approach bias scores did not correlate with current chocolate craving or hunger before or after the task (all *r*s between − 0.143 and 0.023, *p*s > 0.276), but correlated negatively with trait chocolate craving (*r* = − 0.361, *p* = 0.005).

### Conclusion

Study 2 replicated the *trial type* × *stimulus* interaction and, thus, the approach bias towards food found in Study 1. Importantly, Study 2 provided additional mechanistic insights: the differentiation between decision time and grabbing time indicated that the main effect of *stimulus* for grabbing time found in Study 1 might be attributed to the fact that participants were faster to recognize or categorize the food pictures than the objects pictures. Therefore, they start their motor movements in response to food earlier, regardless of where the stimulus is located. Thus, the approach bias found in Study 1 and Study 2 is restricted to the actual grab movement and—in contrast to the finding by Rotteveel and Phaf (2004)—is not reflected in the action preparation stage.

Adding the zooming feature did not affect reaction times as a function of trial type and/or stimulus category. Instead, main effects of the zooming feature emerged for decision and grabbing times that are hard to interpret (as these trial phases should not be affected by zooming). It may be speculated that these effects could be due to a slightly longer inter-trial interval in the zoom group (because of the picture fade-out after reaching the border of the screen). Finally, in contrast to Study 1, dragging time approach bias scores were not correlated with state chocolate craving, and even negatively related to trait chocolate craving.

## Study 3

Study 2 replicated the approach bias towards food as reflected in grab movements and no approach bias as reflected in drag movements. Yet, it might still be that the lack of finding an approach bias towards food when dragging the pictures on the screen may be because participants did not associate the executed arm movements with approaching and avoiding the stimuli. Therefore, we aimed to ensure that participants actually perceive the required movements as approach and avoidance behavior in Study 3. In the literature, this has been achieved by explicitly labeling responses as *towards* and *away* from oneself (Eder & Rothermund, 2008). In fact, it has been found that response patterns can even be reversed by changing response labels. For example, Seibt, Neumann, Nussinson, and Strack (2008) found opposite compatibility effects when using inverse instructions regarding self- versus object-related reference points. Therefore, to exclude the possibility that the lack of an approach bias in dragging time might be because participants did not perceive the downward dragging as moving the stimuli towards them and the upward dragging as moving the stimuli away from them, we manipulated self-reference in Study 3.

For this, we used a between-subjects design where one group of participants performed the task as in Study 1 but, additionally, a manikin representing the participant and the participant’s first name were displayed at the bottom of the screen. As this clearly labeled downward dragging as moving the stimuli towards oneself and upward dragging as moving the stimuli away from oneself, this manipulation was expected to facilitate an approach bias towards food reflected in dragging time. Another group of participants performed the task with the manikin and name displayed at the top of the screen, which was expected to reverse response patterns (Seibt et al., 2008). This group allowed investigating which type of distance cue would dominate approach bias: if the physical location of a target stimulus at the distal side of the screen speeded responses to food in pull trials despite moving the food away from the symbolic self, a dominance of physical over symbolic cues can be inferred. Finally, a control group of participants performed the task as in Study 1, that is, without the manikin and name displayed on the screen.

### Methods

*Participants* Ninety-four individuals (74.5% female, *n* = 70) participated in this study at the University of Salzburg, Austria. Mean age was 23.4 years (SD = 4.74) and mean body mass index was 22.8 kg/m^2^ (SD = 4.30). Most participants had German (55.3%, *n* = 52) or Austrian (36.2%, *n* = 34) citizenship (8.51% other citizenship, *n* = 8). Mean food deprivation was 3.11 h (SD = 2.94).

*AAT* The task and apparatus were equal to Study 1, except that one group of participants performed the AAT with a manikin and the participant’s first name displayed at the bottom of the screen. Another group of participants performed the AAT with the manikin and name displayed at the top of the screen. A third group of participants performed the AAT without the manikin and name (i.e., the same task version as in Study 1 and as the no zoom group in Study 2; Fig. 2c). Participants in the two manikin groups were instructed that the person symbol represented themselves (“A person symbol will be displayed at the top/bottom of the screen. This is you!”). That is, in the group with the manikin at the bottom, the instruction to move the target stimulus towards or away from themselves corresponded to the actual position of the participant in front of the touchscreen monitor. In the group with the manikin at the top, however, this was reversed: the instruction to move the target stimulus towards themselves now corresponded to dragging the stimuli to the top of the screen and the instruction to move the stimulus away from themselves now corresponded to dragging the stimuli to the bottom of the screen.

*Questionnaires* As in Study 1 and Study 2, the chocolate version of FCQ–T–r was used to measure trait chocolate craving (*α* = 0.929), and the chocolate version of the FCQ–S was used to measure current chocolate craving (*α* = 0.897 before and *α* = 0.923 after the task) and hunger (*α* = 0.883 before and *α* = 0.910 after the task).

*Procedure* The study was approved by the institutional review board of the University of Salzburg. Participants were recruited and tested at the University of Salzburg. In the laboratory testing session, participants signed informed consent and completed the FCQ–S. They were then randomly assigned to the AAT either with the manikin at the bottom, with the manikin at the top, or without the manikin. After the AAT, they completed the FCQ–S again as well as the FCQ–T–r and other questionnaires that are not reported here. Participation was reimbursed with course credits.

*Data analyses* Participants in the group with the manikin at the bottom (*n* = 31), the group with the manikin at the top (*n* = 32), and the group without the manikin (*n* = 31) did not differ in sex (*χ*_(2)_^2^ = 1.17, *p* = 0.585), food deprivation, age, trait chocolate craving, or current chocolate craving or hunger before the task (all *F*s < 1.73, *p*s > 0.182). Trials in which participants lifted their hand too early or reached to the wrong picture were excluded from analyses (4.37% of all trials). Bootstrapped split-half reliability estimates in the four conditions (pull food, push food, pull objects, push objects) were *r* = 0.98–0.99 (*r*_sb_ = 0.99) for decision time, *r* = 0.97–0.98 (*r*_sb_ = 0.98–0.99) for grabbing time, and *r* = 0.98–0.99 (*r*_sb_ = 0.99) for dragging time. Median reaction times were submitted to analyses of variance for repeated measures with *group* (manikin at the bottom vs. manikin at the top vs. no manikin) as between-subjects factor, and *trial type* (pull vs. push) and *stimulus* (food vs. objects) as within-subjects factors. Approach bias scores were calculated as in Study 1 and Study 2. Reliability estimates using the *difference*-*of*-*difference* function of *splithalf* were *r* = 0.03 (*r*_sb_ = 0.04) for the decision time approach bias score, *r* = 0.56 (*r*_sb_ = 0.72) for the grabbing time approach bias score, and *r* = 0.59 (*r*_sb_ = 0.72) for the dragging time approach bias score.

### Results

*Decision time* A significant main effect of *stimulus* [*F*_(1,91)_ = 10.9, *p* = 0.001, *η*_p_^2^ = 0.107] indicated that participants reacted faster to food (*M* = 312 ms, SD = 168) than to objects (*M* = 347 ms, SD = 186). The three-way interaction *group *×* trial type *×* stimulus* was also significant [*F*_(2,91)_ = 3.91, *p* = 0.023, *η*_p_^2^ = 0.079]. Following up this interaction by comparing approach bias scores between groups revealed that approach bias scores were higher in the group with the manikin at the bottom (*M* = 6.06 ms, SD = 30.8) than in the group with the manikin at the top (*M* = − 14.2 ms, SD = 26.3; *t*_(61)_ = 2.81, *p* = 0.007). Approach bias scores in the group without a manikin did not significantly differ from the other two groups (both *t*s < 1.80, *p*s > 0.076). No other effects were significant (all *F*s < 3.69, *p*s > 0.057).

*Grabbing time* As in Study 1 and Study 2, the interaction *trial type* × *stimulus* was significant [*F*_(1,91)_ = 5.38, *p* = 0.023, *η*_p_^2^ = 0.056] and, again, following up this interaction effect with paired *t* tests was inconclusive as grabbing times in pull versus push trials were not significantly different for either stimulus category (both *t*s < 1.30, *p*s > 0.199) and did not differ for food versus objects in either trial type (both *t*s < 1.46, *p*s > 0.149). Similar to Study 2, however, grabbing objects in pull trials was slightly slower than grabbing objects in push trials and this direction reversed for foods: they were grabbed faster in pull than in push trials at a descriptive level (Fig. 3c). Thus, this crossed interaction again points to an approach bias towards food as reflected in grab movements. No other effects were significant (all *F*s < 1.89, *p*s > 0.157).

*Dragging time* A significant main effect of *stimulus* [*F*_(1,91)_ = 4.42, *p* = 0.038, *η*_p_^2^ = 0.046] indicated that participants moved objects (*M* = 434 ms, SD = 121) faster than food (*M* = 453 ms, SD = 164). No other effects were significant (all *F*s < 2.04, *p*s > 0.156).

*Correlates of approach bias scores* Decision time, grabbing time, and dragging time approach bias scores did not correlate with trait chocolate craving, current chocolate craving or hunger before or after the task (all *r*s between − 0.185 and 0.074, *p*s > 0.073).

### Conclusion

Study 3 again replicated the *trial type* × *stimulus* interaction for grabbing time and, thus, the approach bias towards food found in Study 1 and Study 2. Although manipulating self-reference did affect decision times, it did not change approach bias towards food as reflected in grab movements or the absence of approach bias towards food as reflected in drag movements. Approach bias scores were not related to trait or state chocolate craving.

## Additional analyses

As the post hoc tests for following up the interaction effects for grabbing time in Studies 1–3 were inconclusive, we explored whether merging data across studies would provide a more clear-cut picture. For this, we merged grabbing times of Study 2 and Study 3 (as grabbing time in Study 1 included decision time), leading to a combined sample size of *n* = 154. An analysis of variance for repeated measures again yielded a significant interaction *trial type* × *stimulus* [*F*_(1,153)_ = 11.5, *p* = 0.001, *η*_p_^2^ = 0.070]. Paired *t* tests indicated that grabbing food (*M* = 478 ms, SD = 199) was faster than grabbing objects (*M* = 498 ms, SD = 219) in pull trials [*t*_(153)_ = 2.09, *p* = 0.038]. Grabbing times for food and objects did not differ in push trials [*t*_(153)_ = 0.67, *p* = 0.504].

## Discussion

The aim of the current studies was to develop a paradigm for measuring approach–avoidance tendencies towards food with arm movements on a touchscreen. Across all three studies, an approach tendency towards food (relative to non-edible objects) was found when participants had to reach towards the stimuli. Specifically, when stimuli were located distally—that is, when participants had to reach out to them in preparation to move the stimuli towards them—there was a speeding of grabbing food compared to non-edible objects. No such approach bias was found for the speed of dragging the stimuli towards or away from oneself. Thus, results differ from conventional tasks that measure approach or avoidance biases by requiring participants to move a manikin or the stimuli on a computer screen (de Houwer et al., 2001; Rinck & Becker, 2007). However, they are in line with findings from a virtual reality study in which an approach bias towards food was reflected in grasping movements towards stimuli (Schroeder et al., 2016).

### Decision time

Due to the fine-grained measurement of a composite, multi-stage, approach–avoidance behavior, our study series gives insights beyond demonstrating an approach bias towards food. First, the present setup allowed for differentiating decision time—that is, the time between stimulus onset and start of the hand movement (release of the start button)—from the subsequent two movement stages grabbing and dragging. In contrast to the findings by Rotteveel and Phaf (2004), those ‘planning times’ (implicated in our decision time) did not carry a bias that would point to a facilitated preparation of affectively compatible movements. As indicated by stimulus type main effects, however, decision times were faster for foods compared to objects in Study 2 and Study 3 (irrespective of trial type). One reason for this might be a higher degree of attentional capture of appetitive food relative to other stimuli (Carbine et al., 2018). Yet, as we did not measure attentional processes (e.g., eye movements) directly, differences in the physical characteristics of food and objects pictures—although those were well-matched—cannot be fully excluded. Another reason may be ‘classification speed’: because participants were instructed to react to either food or non-edible objects, they had to categorize the pictures to identify the target stimuli and execute the required movement. Using such instructions typically leads to faster response latencies to food versus neutral stimuli in simple reaction time tasks because the food category is more specific than the more diverse category of neutral objects (Loeber, Grosshans, Herpertz, Kiefer, & Herpertz, 2013; Meule et al., 2014).

### Grabbing and dragging time

When differentiating between decision and grabbing time, Study 2 and Study 3 converged in showing that approach bias towards food only emerged during grabbing motor movements and not during dragging. Facilitated grabbing on the background of comparable decision and dragging times suggest that stimulus–response compatibility effects potentially driven by approach biases emerge during motoric control of grab movements. Future research might follow-up on this in other AAT implementations, for example, in balance board or virtual reality studies (Eerland et al., 2012; Schroeder et al., 2016) or other setups (e.g., computer gaming, 3D navigation). Dragging was not modulated by stimulus or trial type, suggesting that ‘securing’ food from a distal to a proximal position is not particularly biased, at least in our setup. Yet, the very high internal reliabilities suggest that there was not much variation in dragging times across trials. Likely, participants operated at maximal speed in all trials, which may have concealed stimulus and trial type effects.

### Correlates of approach bias scores

When examining correlates of approach biases, however, no consistent associations were obtained. Grabbing time approach bias did not correlate with trait and state chocolate craving and although dragging time approach bias did correlate with these measures in Study 1, these associations did not replicate in Study 2 and Study 3. One reason for finding no or inconsistent correlations with approach bias scores may be their insufficient reliability. In the current studies, decision time approach bias scores were unreliable. While internal reliability estimates were generally low for grabbing time and dragging time approach bias scores as well, some were in the acceptable range (Parsons, Kruijt, & Fox, 2018). Similarly, it appears that findings on the relationship between approach biases and craving are rather inconclusive. For example, either trait food craving (Brockmeyer et al., 2015), state food craving (Lender et al., 2018), or increases in craving during performing an AAT (Dickson, Kavanagh, & MacLeod, 2016) have been reported to correlate with an approach bias towards food. When looking at the wider literature that include studies using alcohol-, tobacco-, and cannabis-related AATs, relationships between approach biases and craving have also been found inconsistently (Cousijn, Goudriaan, & Wiers, 2011; Schoenmakers, Wiers, & Field, 2008; Wiers et al., 2013, 2014). Thus, future studies are needed that clarify whether approach bias towards food can be found independent of food craving or whether it rather relates to trait, state, or changes in (i.e., cue-induced) food craving.

### Future directions

Interpretation of results is limited to the stimuli and participant characteristics in the current study. That is, results may be different for other stimulus categories (e.g., other appetitive stimuli such as savory foods or alcoholic beverages) and in other samples (e.g., clinical samples such as individuals with eating disorders or obesity). Moreover, future studies need to examine whether approach bias towards food as reflected in grab movements in our paradigm relates to actual consumption of these foods.

An important next step would be to evaluate whether modifying the current paradigm to a training (e.g., by consistently presenting food stimuli at the bottom and control stimuli at the top) results in decreased approach bias and intake of the avoided foods. Previous findings on modifying behavior using approach–avoidance trainings have been ambiguous. For example, while joystick-based trainings (e.g., repeatedly avoiding appetitive stimuli in terms of push movements) have been found to reduce approach tendencies towards appetitive stimuli, effects on actual intake are less consistent (Becker, Jostmann, & Holland, 2018; Kakoschke, Kemps, & Tiggemann, 2017). That is, several studies did not find that an avoidance training reduced actual consumption of alcoholic beverages (Leeman et al., 2018), soft drinks (Krishna & Eder, 2018), or high-calorie foods (Becker, Jostmann, Wiers, & Holland, 2015; Dickson et al., 2016; Ferentzi et al., 2018; Warschburger, Gmeiner, Morawietz, & Rinck, 2018). Among other explanations, one reason may be that these tasks do not involve naturalistic approach and avoidance behaviors, which may hinder translation of training effects into real-world behavior. Therefore, future studies may examine whether touchscreen-based approach–avoidance trainings may be more effective for modifying eating behavior. Such trainings would then need to be rigorously pitted against conventional techniques to reveal the best practices to modify consumption behaviors with approach–avoidance interventions.

## Electronic supplementary material

Below is the link to the electronic supplementary material.

Supplementary material 1 (SAV 24 kb)

Supplementary material 2 (SAV 19 kb)

Supplementary material 3 (SAV 25 kb)
